# Associations between nutrition knowledge and anthropometric indicators in a pre-professional health population: a cross-sectional study

**DOI:** 10.1186/s12909-026-09832-1

**Published:** 2026-07-03

**Authors:** Nezihe Otay Lule, Ferhan Celik, Cagdas Salih Meric, Enes Bahadır Kilic

**Affiliations:** Nutrition and Dietetics Department, Faculty of Health Sciences, Gaziantep University, Gaziantep, Türkiye

**Keywords:** Nutrition knowledge, Anthropometric measurements, Body composition, Bioelectrical impedance analysis, Midwifery students

## Abstract

**Background:**

Anthropometric indicators are widely used to assess nutritional status and obesity-related health risks; however, body mass index (BMI) alone may inadequately reflect central adiposity. Nutrition knowledge has been associated with healthier dietary behaviors, yet evidence linking detailed anthropometric and body composition measures with nutrition knowledge remains limited, particularly among pre-professional health students. This study aimed to examine the associations between general nutrition knowledge and anthropometric and body composition indicators among midwifery students.

**Methods:**

This cross-sectional study included 126 first-year female midwifery students (mean age: 19.83 ± 1.08 years). Anthropometric measurements (BMI, waist circumference [WC], waist-to-hip ratio [WHR], waist-to-height ratio [WHtR], and neck circumference [NC]) and body composition parameters were assessed using standardized procedures and bioelectrical impedance analysis. Nutrition knowledge was evaluated using the General Nutrition Knowledge Questionnaire–Revised (GNKQ-R). Pearson correlation, group comparisons (t-test/ANOVA), and multivariable linear regression analyses were performed. Subscale analyses were adjusted for multiple testing using the Benjamini-Hochberg false discovery rate method.

**Results:**

Higher GNKQ-R total scores were significantly associated with lower BMI (*r* = − 0.27), WC (*r* = − 0.36), WHR (*r* = − 0.23), WHtR (*r* = − 0.36), and body fat percentage (BF%) (*r* = − 0.25), and with higher fat-free mass percentage (all *p* < 0.05). Among GNKQ-R subscales, Healthy food choices and Diet–disease relationships demonstrated the most consistent inverse associations with adiposity-related measures. Group-based analyses showed progressively lower nutrition knowledge scores across increasing WC, WHtR, and body fat risk categories. In multivariable models adjusted for age and income–expense status, WHtR demonstrated the largest independent inverse association with GNKQ-R total score (β = −0.339, *p* < 0.001). BMI and BF% were also independently associated, although with lower explanatory power.

**Conclusion:**

Among midwifery students, higher nutrition knowledge was associated with more favorable anthropometric and body composition profiles, particularly indicators of central adiposity. Within this relatively homogeneous cohort of young, predominantly normal-weight female students, WHtR showed the strongest association with nutrition knowledge among the anthropometric indicators assessed. These findings should be interpreted in light of the cross-sectional design and the limited range of behavioral and sociodemographic variables assessed. Further research is needed to clarify the underlying mechanisms and to determine whether similar associations are observed in more diverse student populations.

**Supplementary Information:**

The online version contains supplementary material available at 10.1186/s12909-026-09832-1.

## Introduction

Anthropometric measurements are among the fundamental and accessible tools used to assess individuals’ nutritional status and obesity-related health risks. Indicators such as body mass index (BMI), waist circumference (WC), waist-to-hip ratio (WHR), waist-to-height ratio (WHtR), neck circumference (NC) and body fat percentage (BF%) are widely employed in the evaluation of obesity and related cardiometabolic risk. However, due to the limitations of BMI in reflecting body fat distribution and central adiposity, waist-based measures -particularly WC and WHtR- have been emphasized as more sensitive indicators of early health risk [1].

Nutrition knowledge is considered an important determinant of healthy dietary behaviors and plays a key role in reducing long-term obesity-related chronic disease risk. Individuals with higher levels of nutrition knowledge are more likely to make healthier food choices and to exhibit more favorable body composition profiles [2]. Nevertheless, BMI has well-recognized limitations in capturing central adiposity and early cardiometabolic risk patterns across populations [1]. At the same time, evidence linking nutrition knowledge with detailed anthropometric and body composition indicators remains limited, particularly among pre-professional health populations.

Midwifery students represent a critical subgroup of future health professionals who will directly influence maternal and infant health during pregnancy, childbirth, and the postpartum period. Evaluating both the nutrition knowledge levels and personal health risk profiles of midwifery students is therefore essential, not only for the protection of their own health but also for their capacity to provide effective nutrition-related guidance in clinical practice. Previous studies suggest that assessing and strengthening nutrition knowledge among future health professionals may contribute to the development of preventive health services and risk-oriented interventions [3].

Accordingly, the aim of this study was to assess anthropometric measurements, body composition indicators, and general nutrition knowledge levels among midwifery students, and to examine the associations between these parameters. The findings are expected to provide a scientific basis for planning evidence-based, risk-oriented nutrition and lifestyle interventions targeting midwifery students and to support the development of preventive health policies.

## Methods

### Study design

This cross-sectional study was conducted between February and May 2025 at the Anthropometry Laboratory of the Department of Nutrition and Dietetics, Gaziantep University, Türkiye. The target population consisted of first-year midwifery students enrolled in the Faculty of Health Sciences, Gaziantep University, during the 2024–2025 academic year.

### Study population and sample size

The study population comprised 134 first-year midwifery students. Sample size calculation was performed using G*Power version 3.1, based on the assumption that the primary analysis would involve a Pearson correlation between GNKQ-R total score and BF%. Assuming a two-tailed hypothesis, an alpha level of 0.05, a statistical power of 80%, and a moderate effect size (*r* = 0.25), the minimum required sample size was calculated as *n* = 124.

A total of 126 students were included in the study, corresponding to a coverage rate of 94% of the target population, thereby exceeding the minimum required sample size. Eight eligible students declined participation or had incomplete data.

### Inclusion and exclusion criteria

Inclusion criteria for the study were being a first-year student enrolled in the Department of Midwifery, being 18 years of age or older, and willingness to participate voluntarily in the study. Exclusion criteria included enrollment in departments other than midwifery, pregnancy or breastfeeding, refusal to participate, the presence of physical or mental conditions that could interfere with anthropometric measurements (such as pacemakers, metal implants, or amputations), and missing or incomplete data.

### Data collection

Data were collected through face-to-face administration of structured questionnaires by trained researchers. Nutritional knowledge was assessed using the General Nutrition Knowledge Questionnaire-Revised (GNKQ-R). Sociodemographic characteristics and anthropometric measurements, including height (cm), waist circumference (WC, cm), hip circumference (cm), waist-to-hip ratio (WHR), waist-to-height ratio (WHtR), neck circumference (NC, cm), and body composition parameters assessed via bioelectrical impedance analysis (BIA), were recorded using a structured data collection form, which is provided as Supplementary File 1. Participants were asked to self-report their perceived income–expense balance and were categorized as having income less than expenses, income equal to expenses, or income greater than expenses. This variable was included as a general indicator of perceived socioeconomic status.

### Assessment of nutritional knowledge

Nutritional knowledge was assessed using the General Nutrition Knowledge Questionnaire–Revised (GNKQ-R). The original General Nutrition Knowledge Questionnaire (GNKQ) was developed by Parmenter and Wardle in 1999 and was subsequently adapted into Turkish, demonstrating acceptable validity and reliability among university students [4]. In response to advances in nutrition science and updates in dietary guidelines and diet–disease relationships, the instrument was revised by Kliemann et al. in 2016, resulting in the GNKQ-R [5]. The Turkish validity and reliability of the revised GNKQ-R were confirmed in 2022 [6].

The GNKQ-R consists of 48 questions comprising 88 items across four subscales:

- GNKQ-R1: Nutrition recommendations (9 questions; items 1–18; maximum score: 18)
- GNKQ-R2: Food groups (10 questions; items 19–54; maximum score: 36)
- GNKQ-R3: Healthy food choices (13 questions; items 55–67; maximum score: 13)
- GNKQ-R4: Diet-disease relationships (16 questions; items 68–88; maximum score: 21).

The subscales assess, respectively, participants’ knowledge of official dietary recommendations; their understanding of food groups and the primary nutrients contained in foods; their ability to make healthier food choices in everyday situations; and their awareness of the relationships between dietary patterns and chronic diseases.

Each item is scored as correct (1 point) or incorrect (0 points). Subscale scores are calculated as the sum of correct responses within each domain, yielding a total possible score ranging from 0 to 88. Higher scores indicate greater nutritional knowledge.

### Anthropometric measurements

All measurements were performed and categorized in accordance with international guidelines. Height was measured using a stadiometer with participants standing in the Frankfort plane and with feet positioned side by side [7]. BMI was calculated as body weight divided by the square of height and classified accordingly [8]. WC was measured at the midpoint between the lowest rib and the iliac crest; values ≥ 80 cm were considered “at risk” and ≥ 88 cm as “high risk” [9]. Hip circumference was measured at the widest part of the hips, parallel to the floor. A WHR > 0.85 was accepted as indicative of increased risk for chronic diseases [9]. WHtR, an age- and sex-independent indicator used to assess abdominal obesity risk, was calculated by dividing WC by height; values ≥ 0.5 were classified as “increased health risk” and ≥ 0.6 as “substantially increased health risk” [10]. NC, a measure of upper-body fat distribution, was assessed using a non-elastic measuring tape placed just below the cricothyroid cartilage with the head positioned in the Frankfort plane. Values ≥ 34 cm were considered indicative of obesity and cardiometabolic risk [11].

### Body composition assessment

Body composition was assessed using bioelectrical impedance analysis (BIA), a safe, non-invasive, and cost-effective method widely used in clinical practice. BIA estimates body composition by measuring the resistance of body tissues to a low-level electrical current, with adipose tissue demonstrating lower conductivity compared to lean tissue [12, 13]. Measurements were performed using a multifrequency bioelectrical impedance analyzer (X-Contact 350, JAWON Medical Co., Seoul, Republic of Korea) located at the Department of Nutrition and Dietetics, Gaziantep University. Participants were measured in a fasting state, standing upright, barefoot, and without metal accessories. For all participants, measurements were conducted outside the menstrual period to minimize fluid-related variability. The analysis provided estimates of body weight (kg), BMI; kg/m², BF%, fat-free mass percentage (FFM%), total body water (TBW), extracellular water (ECW) and basal metabolic rate (BMR).

BF% was classified based on sex-specific reference ranges. According to the reference values proposed by Gallagher et al. for women, values between 21 and 32% were considered normal, 33–37% were classified as increased, and a BF% of ≥ 38% was regarded as indicative of obesity-related high adiposity [14].

### Statistical analysis

Statistical analyses were performed using the Statistical Package for the Social Sciences (SPSS), version 22.0 (IBM Corp., Armonk, NY, USA). Descriptive statistics were presented as mean ± standard deviation (SD) for continuous variables and as number (n) and percentage (%) for categorical variables. The normality of continuous variables was assessed using the Shapiro–Wilk test. Comparisons of mean values between two independent groups were conducted using the independent samples t-test, while comparisons involving three or more groups were performed using one-way analysis of variance (ANOVA). When ANOVA indicated a statistically significant difference, post-hoc pairwise comparisons were carried out using the Games–Howell test, as appropriate. Associations between continuous variables were examined using Pearson correlation analysis.

Subscale analyses of the GNKQ-R were conducted for anthropometric and body composition indicators that demonstrated significant associations with the GNKQ-R total score. To control for multiple testing across GNKQ-R subscales, *p*-values were adjusted using the Benjamini–Hochberg false discovery rate method.

To identify independent predictors of general nutrition knowledge, multivariable linear regression analyses were performed with the GNKQ-R total score as the dependent variable. A baseline model including age and income–expense status was first constructed. Subsequently, separate models were fitted to evaluate the independent associations of WHtR, BMI, and BF%, each adjusted for age and income–expense status. All regression models were estimated using the enter method. Multicollinearity was assessed using variance inflation factors (VIF), with values < 5 considered acceptable. All statistical tests were two-tailed, and a *p*-value < 0.05 was considered statistically significant.

## Results

A total of 126 first-year midwifery students were included in the study, corresponding to a coverage rate of 94% of the target population (*n* = 134). The mean age of the participants was 19.83 ± 1.08 years (range: 18–24), and all participants were female. With regard to income–expense status, the majority reported balanced income and expenses (69.0%), while 24.6% reported income lower than expenses.

Based on BMI classification, 64.3% of participants were of normal weight, 13.5% were underweight, 20.6% were overweight, and 1.6% were classified as obese. According to central and regional adiposity indicators, most participants were categorized as having no cardiometabolic risk, although increased risk was observed in 14.3% for WC, 11.1% for WHtR, and 11.1% for NC. Only 1.6% of participants were classified as at risk based on WHR.

Mean anthropometric measurements were 73.13 ± 7.01 cm for WC, 0.74 ± 0.05 for WHR, 0.45 ± 0.04 for WHtR, and 31.68 ± 1.65 cm for NC. The mean BMI was 22.51 ± 3.61 kg/m². Body composition analysis revealed a mean BF% of 27.01 ± 5.81 and a mean FFM% of 72.99 ± 5.81. Mean TBW was 31.21 ± 3.32 L, with an ECW/TBW ratio of 0.39 ± 0.03. The average BMR was 1281.96 ± 53.88 kcal/day.

The mean GNKQ-R total score was 45.72 ± 9.64. Among subscales, mean scores were 7.63 ± 1.80 for nutrition recommendations, 18.23 ± 4.61 for food groups, 7.26 ± 2.49 for healthy food choices, and 12.58 ± 3.65 for diet–disease relationships (Table 1).

**Table 1:** Descriptive characteristics, anthropometric measurements, body composition parameters, and GNKQ-R scores of the study population

Characteristics (*n*:126)	Variable	*n* (%)
Age (years)	*Mean ± SD*	19.83 ± 1.08 (18–24)
Sex	Female	126 (100.0)
Income-expense status	Income **<** expenses	31 (24.6)
	Income **=** expenses	87 (69.0)
	Income **>** expenses	8 (6.3)
BMI classification	Underweight	17 (13.5)
	Normal weight	81 (64.3)
	Overweight	26 (20.6)
	Obese	2 (1.6)
WC	No risk	102 (81.0)
	Increased risk	18 (14.3)
	High risk	6 (4.8)
WHR	No risk	124 (98.4)
	At risk	2 (1.6)
WHtR	No risk	112 (88.9)
	Increased risk	14 (11.1)
NC	No risk	112 (88.9)
	Increased risk	14 (11.1)
BF%	Low	22 (17.5)
	Normal	80 (63.5)
	Increased	24 (19.0)
		**Mean ± SD (min–max)**
Anthropometric measurements	WC *(cm)*	73.13 ± 7.01 (61.0–95.0)
	WHR	0.74 ± 0.05 (0.65–0.90)
	WHtR	0.45 ± 0.04 (0.37–0.57)
	NC *(cm)*	31.68 ± 1.65 (27.0–36.0)
Body composition parameters *(BIA)*	BMI *(kg/m²)*	22.51 ± 3.61 (16.09–33.65)
	BF%	27.01 ± 5.81 (15.20–40.28)
	FFM *(%)*	72.99 ± 5.81 (59.72–84.80)
	TBW*(L)*	31.21 ± 3.32 (23.8–40.5)
	ECW*(L)*	12.31 ± 1.36 (9.5–16.0)
	ECW/TBW	0.39 ± 0.03 (0.38–0.41)
	BMR *(kcal/day)*	1281.96 ± 53.88 (1150–1439)
GNKQ-R scores	GNKQ-R1 *Nutrition recommendations*	7.63 ± 1.80 (4–11)
	GNKQ-R2 *Food groups*	18.23 ± 4.61 (6–28)
	GNKQ-R3 *Healthy food* *choices*	7.26 ± 2.49 (1–11)
	GNKQ-R4 *Diet-disease reslationship*	12.58 ± 3.65 (0–18)
	GNKQ-R Total	45.72 ± 9.64 (25–63)

All continuous variables showed normal distribution. Anthropometric and body composition risk classifications were performed based on sex-specific cut-off values recommended by international guidelines

*Abbreviations*: *WC* waist circumference, *WHR* waist-to-hip ratio, *WHtR* waist-to-height ratio, *NC* neck circumference, *BF%* body fat percentage, *FFM%* fat-free mass percentage, *TBW* total body water, *BMR* basal metabolic rate, *ECW/TBW* extracellular water to total body water ratio, *GNKQ-R* General Nutrition Knowledge Questionnaire–Revised, *BIA* bioelectrical impedance analysis

Pearson correlation analysis showed that the GNKQ-R total score was significantly and inversely correlated with several anthropometric and body composition parameters. Higher nutritional knowledge was associated with lower BMI (*r* = − 0.27, *p* < 0.05), WC (*r* = − 0.36, *p* < 0.001), WHR (*r* = − 0.23, *p* < 0.05), WHtR (*r* = − 0.36, *p* < 0.001), and BF% (*r* = − 0.25, *p* < 0.05). Conversely, a positive correlation was observed between GNKQ-R total score and FFM% (*r* = 0.25, *p* < 0.05).

Subscale analyses revealed that the “Healthy food choices” and “Diet-disease relationship” subscales demonstrated the most consistent associations with adiposity-related measures. WC and WHtR were negatively correlated with all GNKQ-R subscales. Age and NC were not significantly correlated with the GNKQ-R total score or subscale scores. Weak inverse associations were also observed between the “Healthy food choices” and “Diet-disease relationship” subscales and selected body composition-related parameters, including TBW, ECW/TBW, and BMR (Table 2).

**Table 2:** Correlations between GNKQ-R scores and anthropometric and body composition parameters

Variable (*n*:126)	GNKQ-*R* Total	GNKQ-R1 Nutrition recommendations	GNKQ-R2 Food groups	GNKQ-R3 Healthy food choices	GNKQ-R4 Diet-disease relationship
Age	−0.15	−0.13	−0.09	−0.14	−0.12
BMI *(kg/m²)*	−0.27*	−0.16	−0.10	−0.29*	−0.31**
WC *(cm)*	−0.36**	−0.18*	−0.22*	−0.35**	−0.33**
WHR	−0.23*	−0.15	−0.14	−0.27*	−0.16
WHtR	−0.36**	−0.18*	−0.23*	−0.35**	−0.32**
NC *(cm)*	−0.16	−0.02	−0.16	−0.17	−0.08
BF%	−0.25*	−0.17	−0.12	−0.25*	−0.26*
FFM%	0.25*	0.17	0.12	0.25*	0.26*
TBW	−0.17	−0.09	−0.03	−0.21*	−0.23*
ECW/TBW	−0.11	0.02	−0.01	−0.19*	−0.15
BMR *(kcal/day)*	−0.17	−0.08	−0.04	−0.20*	−0.20*

Pearson correlation coefficients (r) are presented. *p < 0.05, **p < 0.001

*Abbreviations*: *WC* waist circumference, *WHR* waist-to-hip ratio, *WHtR* waist-to-height ratio, *NC* neck circumference, *BF%* body fat percentage, *FFM%* fat-free mass percentage, *TBW* total body water, *BMR* basal metabolic rate, *ECW/TBW* extracellular water to total body water ratio, *GNKQ-R* General Nutrition Knowledge Questionnaire–Revised

The comparison of GNKQ-R total and subscale scores across anthropometric risk classifications is presented in Table 3. Overall, higher anthropometric risk status was associated with lower nutrition knowledge scores, although the magnitude and pattern of associations varied across anthropometric indices and GNKQ-R subdomains.

**Table 3:** Comparison of GNKQ-R total and subscale scores across anthropometric risk classifications

Anthropometric risk classification	GNKQ-*R* Total	GNKQ-R1 Nutrition recommendations	GNKQ-R2 Food groups	GNKQ-R3 Healthy food choices	GNKQ-R4 Diet-disease relationship
BMI	F = 3.69, ***p* = 0.014**	F = 1.36 *p* = 0.259	F = 0.61 *p* = 0.608	F = 4.37 ***p*** **= 0.006**	F = 4.60 ***p*** **= 0.004**
Underweight	48.65 ± 8.69	7.94 ± 1.30	18.94 ± 5.49	8.00 ± 2.21	13.76 ± 2.39
Normal	46.85 ± 8.90	7.75 ± 1.90	18.42 ± 4.51	7.60 ± 2.42	13.07 ± 3.44
Overweight/Obese	40.68 ± 10.75	7.11 ± 1.73	17.25 ± 4.39	5.86 ± 2.41	10.46 ± 4.13
WC	F = 15.79 ***p*** **< 0.001**	F = 5.63 ***p*** **= 0.005**	F = 4.44 ***p*** **= 0.014**	F = 15.69 ***p*** **< 0.001**	F = 13.36 ***p*** **< 0.001**
No risk	47.71 ± 8.53	7.88 ± 1.76	18.75 ± 4.74	7.80 ± 2.23	13.26 ± 3.13
Increased risk	39.22 ± 9.56	6.72 ± 1.67	16.67 ± 3.45	5.28 ± 2.32	10.56 ± 3.91
High risk	31.50 ± 8.29	6.17 ± 1.60	14.00 ± 1.67	4.17 ± 2.23	7.17 ± 4.83
BF%	F = 9.04, ***p* < 0.001**	F = 3.65 ***p*** **= 0.015**	F = 3.03 ***p*** **= 0.032**	F = 5.46 ***p*** **= 0.001**	F = 9.53 ***p*** **< 0.001**
Low	47.91 ± 7.90	7.91 ± 1.34	18.41 ± 4.99	8.00 ± 2.07	13.59 ± 2.34
Normal	47.65 ± 8.83	7.88 ± 1.91	18.90 ± 4.72	7.59 ± 2.32	13.29 ± 3.34
Increased	37.29 ± 9.60	6.58 ± 1.49	15.83 ± 3.17	5.54 ± 2.78	9.33 ± 4.06
WHR	**t = 1.98** ***p*** **= 0.050**	t = 0.90 *p* = 0.372	t = 0.69 *p* = 0.493	t = 1.59 *p* = 0.114	**t = 2.84** ***p*** **= 0.005**
No risk	45.94 ± 9.56	7.65 ± 1.81	18.27 ± 4.63	7.31 ± 2.47	12.70 ± 3.50
At risk	32.50 ± 6.36	6.50 ± 0.71	16.00 ± 4.24	4.50 ± 3.54	5.50 ± 7.78
WHtR	**t = 4.948** ***p* < 0.001**	**t = 4.155** ***p* < 0.001**	**t = 2.190** ***p* = 0.030**	**t = 4.994** ***p* < 0.001**	**t = 4.012** ***p* = 0.001**
No risk	47.31 ± 8.71	7.84 ± 1.80	18.59 ± 4.69	7.69 ± 2.30	13.19 ± 3.17
Increased risk	36.17 ± 9.67	6.39 ± 1.29	16.06 ± 3.51	4.78 ± 2.21	8.94 ± 4.30
NC	**t = 2.15** ***p*** **= 0.034**	t = 1.72 *p* = 0.087	t = 1.87 *p* = 0.063	t = 1.23 *p* = 0.222	t = 1.58 *p* = 0.117
No risk	46.37 ± 9.63	7.73 ± 1.80	18.50 ± 4.73	7.37 ± 2.47	12.77 ± 3.59
Increased risk	40.57 ± 8.38	6.86 ± 1.75	16.07 ± 2.87	6.50 ± 2.65	11.14 ± 3.92

Values are presented as mean ± standard deviation (SD). For three-group comparisons, F statistics and corresponding p values from one-way ANOVA are reported; for two-group comparisons, independent samples t-test p values are provided. Statistically significant results are shown in bold

*Abbreviations*: *WC* waist circumference, *WHR* waist-to-hip ratio, *WHtR* waist-to-height ratio, *NC* neck circumference, *BF%* body fat percentage, *GNKQ-R* General Nutrition Knowledge Questionnaire–Revised

### Body mass index (BMI)

When participants were classified into three BMI categories (underweight, normal, and overweight/obese), a significant overall difference was observed for the GNKQ-R total score (F = 3.69, *p* = 0.014). Among subscales, significant between-group differences were detected for Healthy food choices (R3) (F = 4.37, *p* = 0.006) and Diet–disease relationship (R4) (F = 4.60, *p* = 0.004), whereas Nutrition recommendations (R1) and Food groups (R2) did not differ significantly across BMI categories. Post-hoc analyses indicated that these differences were primarily driven by lower R3 and R4 scores in the overweight/obese group compared with normal-weight participants.

### Waist circumference (WC)

WC risk classification showed robust and consistent associations with nutrition knowledge. GNKQ-R total scores differed markedly across WC risk groups (F = 15.79, *p* < 0.001). All GNKQ-R subscales were significantly associated with WC categories (R1: F = 5.63, *p* = 0.005; R2: F = 4.44, *p* = 0.014; R3: F = 15.69, *p* < 0.001; R4: F = 13.36, *p* < 0.001). Post-hoc comparisons demonstrated a clear gradient, with progressively lower GNKQ-R total and subscale scores observed from the no-risk group to the high-risk group, particularly pronounced for R3 and R4.

### Body fat percentage (BF%)

Similarly, BF% categories were significantly associated with GNKQ-R total score (F = 9.04, *p* < 0.001). All four subscales differed significantly across BF% groups (R1: F = 3.65, *p* = 0.015; R2: F = 3.03, *p* = 0.032; R3: F = 5.46, *p* = 0.001; R4: F = 9.53, *p* < 0.001). Post-hoc analyses revealed that participants classified into higher BF% risk categories had consistently lower nutrition knowledge scores, with the strongest effects again observed for Healthy food choices and Diet-disease relationship subscales.

### Waist-to-hip ratio (WHR)

In two-group comparisons based on WHR risk status, the GNKQ-R total score was borderline significant (t = 1.98, *p* = 0.050). No significant differences were observed for R1, R2, or R3 subscales, whereas the Diet–disease relationship (R4) subscale showed a significant difference (t = 2.84, *p* = 0.005), indicating lower disease-related nutrition knowledge among participants classified as at risk. These findings should be interpreted with caution due to the very small number of participants in the at-risk WHR category.

### Neck circumference (NC)

NC risk classification was significantly associated with the GNKQ-R total score (t = 2.15, *p* = 0.034). However, none of the GNKQ-R subscales reached statistical significance individually (all *p* > 0.05), suggesting that the observed difference in total score reflects a modest, cumulative effect across domains rather than a single dominant knowledge area.

### Waist-to-height ratio (WHtR)

WHtR showed the most consistent associations with nutrition knowledge across the anthropometric indices examined. Participants classified as at risk based on WHtR had substantially lower GNKQ-R total scores (t = 4.948, *p* < 0.001). Significant differences were also observed across multiple subscales, including Nutrition recommendations (R1) (t = 4.155, *p* < 0.001), Food groups (R2) (t = 2.190, *p* = 0.030), Healthy food choices (R3) (t = 4.994, *p* < 0.001), and Diet-disease relationship (R4) (t = 4.012, *p* = 0.001). Among all anthropometric indices examined, WHtR demonstrated the most consistent and broad-based associations across GNKQ-R domains.

Multivariable Linear Regression Analyses for GNKQ-R Total Score in Table 4. In the baseline model including age and income–expense status, no significant association was observed with GNKQ-R total score (adjusted R² = 0.011).

**Table 4:** Multivariable Linear Regression Analyses for GNKQ-R Total Score

Model 1	Variable	B	SE	β	t	*p*	95% CI for B	VIF
Baseline model	Constant	74.563	15.907	–	4.688	< 0.001	43.074/106.051	NA
	Age	−1.450	0.803	−0.162	−1.806	0.073	−3.039 / 0.139	1.019
	Income< expenses	−1.202	2.008	−0.054	−0.599	0.551	−5.177 / 2.774	1.024
	Income> expenses	3.356	3.577	0.085	0.938	0.350	−3.726/ 10.437	1.042
	**Adjusted R²**:	**0.011**						

Model 2	Variable	B	SE	β	t	p	95% CI for B	VIF
Central adiposity model (WHtR)	Constant	93.067	15.773	–	5.900	< 0.001	61.83 / 124.29	NA
	Age	−0.746	0.781	−0.083	−0.955	0.341	−2.291 / 0.800	1.076
	Income< expenses	−0.910	1.901	−0.041	−0.479	0.633	−4.674 / 2.854	1.026
	Income> expenses	3.555	3.385	0.090	1.050	0.296	−3.146/ 10.255	1.042
	**WHtR**	**−72.161**	**18.439**	**−0.339**	**−3.914**	**< 0.001**	**−108.665/−35.657**	**1.060**
	**Adjusted R²**:	**0.115**						

Model 3	Variable	B	SE	β	t	p	95% CI for B	VIF
General adiposity model (BMI)	Constant	77.186	15.552	–	4.963	< 0.001	46.39 / 107.97	NA
	Age	−0.847	0.815	−0.095	−1.039	0.301	−2.461 / 0.767	1.104
	Income< expenses	−0.102	2.002	−0.005	−0.051	0.959	−4.067 / 3.862	1.070
	Income> expenses	3.449	3.491	0.088	0.988	0.325	−3.463/ 10.360	1.042
	**BMI (kg/m²)**	**−0.660**	**0.247**	**−0.247**	**−2.670**	**0.009**	**−1.149/−0.171**	**1.133**
	**Adjusted R²**:	**0.058**						

Model 4	Variable	B	SE	β	t	p	95% CI for B	VIF
Total adiposity model (BF%)	Constant	74.127	15.587	–	4.756	< 0.001	43.26 / 104.98	NA
	Age	−0.928	0.815	−0.104	−1.139	0.257	−2.541 / 0.685	1.093
	Income< expenses	−0.478	1.990	−0.021	−0.240	0.811	−4.416 / 3.461	1.047
	Income> expenses	3.558	3.506	0.090	1.015	0.312	−3.384/ 10.499	1.042
	**Body fat (%)**	**−0.374**	**0.152**	**−0.225**	**−2.465**	**0.015**	**−0.674/−0.074**	**1.099**
	**Adjusted R²**:	**0.050**						

Values are presented as unstandardized coefficients (B), Std. Error (SE), standardized coefficients (β), and corresponding 95% confidence intervals. All models were estimated using the enter method

Model 1 represents the baseline model including age and income–expense status. Models 2–4 additionally include waist-to-height ratio (WHtR), body mass index (BMI), and body fat percentage (BF%), respectively

Income = expenses was used as the reference category

*GNKQ-R* General Nutrition Knowledge Questionnaire–Revised, *WHtR* waist-to-height ratio, *BMI* body mass index

In multivariable linear regression analyses adjusted for age and income–expense status, WHtR demonstrated the largest independent standardized effect size in relation to GNKQ-R total score (β = −0.339, *p* < 0.001), accounting for 11.5% of the variance. In separate models, both BMI (standardized β = −0.247, *p* = 0.009; adjusted R² = 0.058) and BF% (standardized β = −0.225, *p* = 0.015; adjusted R² = 0.050) were also independently and inversely associated with GNKQ-R total score, although the magnitude of association and explained variance were lower than those observed for WHtR.

Age and income–expense status were not independently associated with GNKQ-R total score in any of the models.

## Discussion

This study indicates that general nutrition knowledge is meaningfully associated with anthropometric and body composition profiles among first-year midwifery students, a pre-professional health population at the very beginning of formal health education. Indicators reflecting central adiposity-particularly WC, WHtR, and BF%-showed consistent associations with nutrition knowledge, whereas age and fluid distribution parameters were not related. Together, these findings indicate statistically significant associations between nutrition knowledge and central adiposity indicators within this cohort. However, given the cross-sectional design, these results do not imply a direct effect of knowledge on fat distribution and should be interpreted as correlational.

The descriptive findings revealed that most participants were classified as having normal body weight according to BMI, a pattern consistent with previous studies among female university students and reflective of the young age and early academic stage of the sample [15]. However, despite this generally favorable BMI profile, a notable proportion of students exhibited increased cardiometabolic risk when assessed using alternative anthropometric indicators. Elevated WC, WHtR, and NC were observed in approximately one-tenth to one-seventh of participants, highlighting a discordance between BMI and measures of central or upper-body adiposity and reinforcing concerns that reliance on BMI alone may underestimate early health risk in young women [16]. From an educational perspective, this finding underscores the importance of equipping future health professionals with a nuanced understanding of anthropometric assessment beyond conventional weight-based indices.

Waist-based measures, particularly WHtR, have been increasingly recognized as sensitive and practical indicators of adiposity-related risk, especially in populations where overt obesity is uncommon [16, 17]. The detection of elevated risk using these indices in a predominantly normal-weight cohort underscores their value for early screening in university settings and supports their use as complementary tools to BMI. Such measures may capture subtle changes in fat distribution that precede clinically apparent obesity [18]. These observations underscore the importance of comprehensive anthropometric assessment in young adult populations, particularly in settings where BMI alone may underestimate early risk patterns.

In terms of nutrition knowledge, the mean GNKQ-R total score indicated a suboptimal level of general nutrition knowledge for a pre-professional health population. While this level may be expected among students at the beginning of their professional education, it also highlights the need for further research examining how nutrition knowledge evolves during early professional training. Variability across GNKQ-R subdomains indicates differential patterns of association across knowledge domains within this cohort.

Correlation analyses demonstrated that higher nutrition knowledge was consistently associated with lower indices of both general and central adiposity, including BMI, WC, WHtR, and BF%, alongside a higher proportion of fat-free mass. Although the magnitude of these correlations was modest, their consistency across multiple indicators supports the relevance of nutrition knowledge even at early stages of professional development.

Among anthropometric measures, WC and WHtR showed the most consistent associations with GNKQ-R total and subscale scores, suggesting that central adiposity indicators demonstrated stronger statistical associations with nutrition knowledge than BMI in this sample. Given that waist-based indices are sensitive markers of early cardiometabolic risk [19, 20], these findings may be consistent with the hypothesis that behavioral factors related to dietary choices and lifestyle co-vary with nutrition knowledge; however, such mechanisms were not directly assessed in the present study. From a biological and behavioral perspective, it is plausible that nutrition knowledge may relate indirectly to adiposity patterns through lifestyle-related pathways. Greater awareness of dietary recommendations and diet–disease relationships may be associated with more informed food choices, portion control, and overall energy balance, which in turn influence fat accumulation and distribution. Central adiposity has been reported to be metabolically responsive to long-term dietary patterns and energy imbalance [21]. However, because dietary intake, physical activity, and other behavioral mediators were not directly measured in the present study, these explanations remain hypothetical and cannot be confirmed within the current dataset. In addition to these behavioral considerations, the differential strength of associations observed between central adiposity indicators and BMI may also reflect measurement-related factors. BMI represents overall body mass relative to height and does not differentiate between fat mass and lean mass, nor does it capture regional fat distribution [1]. In relatively young and homogeneous populations, variability in BMI may therefore be limited, potentially reducing its sensitivity to detect subtle behavior-related differences in adiposity. By contrast, waist-based indices more directly reflect abdominal fat accumulation, which may be more responsive to sustained dietary patterns and energy balance. This distinction may partly explain why WC and WHtR demonstrated stronger statistical associations with nutrition knowledge than BMI in the present sample.

Analysis of GNKQ-R subdomains indicated that Healthy food choices and Diet–disease relationship were the domains most strongly associated with adiposity-related measures. These findings indicate that certain knowledge domains demonstrated stronger statistical associations with anthropometric indicators in this cohort. Similar domain-specific patterns in nutrition knowledge research have been reported previously [22], although causal mechanisms cannot be inferred from the present cross-sectional data.

In contrast, NC was not significantly associated with nutrition knowledge, whereas ECW/TBW showed weak inverse associations with selected applied nutrition knowledge subscales. While NC reflects upper-body subcutaneous fat accumulation [23, 24], its limited association in this cohort may be attributable to the low prevalence of advanced adiposity and the predominantly normal-weight profile of participants. The weak association observed between ECW/TBW and the “Healthy food choices” subscale should be interpreted cautiously, as fluid distribution is influenced by multiple physiological factors beyond dietary awareness [25–27]. Similarly, weak inverse associations between applied nutrition knowledge subscales and BMR were observed, likely reflecting indirect relationships mediated by body composition rather than a direct effect of nutrition knowledge.

Group-based analyses complemented the correlation findings by demonstrating that higher anthropometric risk categories were generally associated with lower nutrition knowledge scores, although the magnitude and pattern of these associations differed across indices. Waist-based indicators again exhibited the most pronounced gradients. This pattern is consistent with previous research suggesting that measures of central adiposity may show closer associations with lifestyle-related factors than BMI [28, 29]. In contrast, BMI demonstrated more limited associations in this cohort, aligning with evidence that it may reflect more generalized or later-stage adiposity rather than earlier distributional changes in body fat [30].

BF% showed consistent differences across all GNKQ-R subscales, indicating that total adiposity is broadly associated with nutrition knowledge. This finding is consistent with previous evidence suggesting that overall body composition parameters have been reported to be associated with nutrition-related knowledge and health behaviors [31, 32].

Taken together, the results presented in Table 3 indicate that waist-based anthropometric measures—particularly WHtR—showed the strongest statistical associations with nutrition knowledge in this sample. Differences across knowledge subdomains further reflect variation in the magnitude of these associations. These findings are consistent with previous reports suggesting that knowledge domains related to healthy food choices and diet–disease relationships have shown comparatively stronger associations with anthropometric risk indicators in young adult populations [31, 33].

Multivariable regression analyses demonstrated that WHtR showed the strongest independent statistical association with GNKQ-R total score after adjustment for age and income–expense status, whereas BMI and BF% showed smaller associations. The absence of significant effects for age and socioeconomic indicators likely reflects the relative homogeneity of the study population. Importantly, because behavioral mediators such as dietary intake and physical activity were not assessed, the observed associations cannot be interpreted as evidence of a direct effect of nutrition knowledge on body composition. Residual confounding and reverse causality therefore remain possible. Future longitudinal studies incorporating behavioral measures are needed to clarify the direction and mechanisms underlying these relationships.

### Limitations

This study has several limitations. The cross-sectional design does not allow causal inferences regarding the relationship between nutrition knowledge and anthropometric or body composition parameters. The study population consisted solely of first-year female midwifery students from a single institution, which may limit the generalizability of the findings to other student groups, male participants, or individuals at later stages of professional training. Nutrition knowledge was assessed using a self-administered questionnaire, and dietary intake and physical activity were not directly measured; therefore, nutrition knowledge may not fully reflect actual dietary behaviors, and the behavioral mechanisms through which nutrition knowledge may influence anthropometric and body composition outcomes could not be evaluated in the present study. The income–expense variable represents a subjective indicator of perceived financial balance and does not directly measure food access, food security, or nutritional adequacy; therefore, its relationship with anthropometric outcomes should be interpreted cautiously. In addition, the analysis included a limited set of sociodemographic variables, and other unmeasured lifestyle and sociodemographic factors—such as parental education, living arrangements, sleep patterns, and psychosocial influences—may have affected the observed associations. Reverse causality cannot be excluded, as individuals with healthier body composition profiles may also be more motivated to acquire nutrition-related knowledge. Finally, the single-center design and lack of longitudinal follow-up limit the ability to assess temporal changes in nutrition knowledge and adiposity-related outcomes.

## Conclusion

This study demonstrates statistically significant associations between nutrition knowledge and anthropometric risk profiles among first-year midwifery students. Measures of central adiposity—particularly WHtR and WC—showed stronger associations with nutrition knowledge than BMI in this cohort. Given the cross-sectional design and the absence of direct behavioral measures, these findings should be interpreted as correlational and do not establish causality. Future longitudinal research incorporating behavioral variables is needed to clarify the direction and mechanisms underlying these associations.

## Supplementary Information

Supplementary Material 1.

## Data Availability

The datasets generated and/or analyzed during the current study are not publicly available due to institutional data protection policies but are available from the corresponding author on reasonable request.

## Abbreviations

BMI: Body Mass Index
WC: Waist Circumference
WHR: Waist-to-Hip Ratio
WHtR: Waist-to-Height Ratio
NC: Neck Circumference
BF%: Body Fat Percentage
FFM%: Fat-Free Mass Percentage
TBW: Total Body Water
ECW: Extracellular Water
BMR: Basal Metabolic Rate
ECW/TBW: Extracellular Water to Total Body Water Ratio
GNKQ-R: General Nutrition Knowledge Questionnaire–Revised
