# Supplementary material for: Associations between nutrition knowledge and anthropometric indicators in a pre-professional health population: a cross-sectional study
Source: BMC Med Educ. 2026 Jul 3;26:1393. doi: 10.1186/s12909-026-09832-1 (PMC13520338; doi:10.1186/s12909-026-09832-1)
Supplement: Supplementary file 1 [file 12909_2026_9832_MOESM1_ESM.pdf]

Supplementary File 1. Structured Data Collection Form

Sociodemographic Characteristics

Date of Birth: …../…../…… (Day/Month/Year)
Sex:
1. Male
2. Female

Marital Status:
1. Married
2. Single

What is your income–expense status?
1. Income is higher than expenses
2. Income equals expenses
3. Income is lower than expenses

Anthropometric Measurements

Height: ……………. cm
Body weight: ……………… kg
Body mass index (BMI): ……………… kg/m²

Waist circumference: …………………… cm
Hip circumference: …………………… cm

Waist-to-hip ratio (WHR): ………………
Waist-to-height ratio (WHtR): ………………
Neck circumference: ……………… cm

Body Composition Analysis

(To be completed after bioelectrical impedance analysis [BIA])

Body fat (%): ………………
Fat-free mass: ………………
Fat mass: ………………
Total body water: ………………
Body water (%): ………………
Metabolic age: ………………
Basal metabolic rate (BMR): ………………
